# An acidic model pro-peptide affects the secondary structure, membrane interactions and antimicrobial activity of a crotalicidin fragment

**DOI:** 10.1038/s41598-018-29444-0

**Published:** 2018-07-24

**Authors:** Nelson G. O. Júnior, Marlon H. Cardoso, Elizabete S. Cândido, Daniëlle van den Broek, Niek de Lange, Nadya Velikova, J. Mieke Kleijn, Jerry M. Wells, Taia M. B. Rezende, Octávio Luiz Franco, Renko de Vries

**Affiliations:** 1 Centro de Análises Proteômicas e Bioquímicas, Programa de Pós-Graduação em Ciências Genômicas e Biotecnologia, Universidade Católica de Brasília, Brasília-DF, Brazil; 2 Physical Chemistry and Soft Matter, Wageningen University, Stippeneng 4, 6708 WE Wageningen, The Netherlands; 3 Programa de Pós-Graduação em Patologia Molecular, Faculdade de Medicina, Universidade de Brasília, Brasília-DF, Brazil; 4 S-inova Biotech, Programa de Pós-Graduação em Biotecnologia, Universidade Católica Dom Bosco, Campo Grande, MS Brazil; 5 Host-Microbe Interactomics, Wageningen University, P.O. Box 338, 6700AH Wageningen, The Netherlands; 6 Curso de Odontologia, Universidade Católica de Brasília, Brasília-DF, Brazil

## Abstract

In order to study how acidic pro-peptides inhibit the antimicrobial activity of antimicrobial peptides, we introduce a simple model system, consisting of a 19 amino-acid long antimicrobial peptide, and an N-terminally attached, 10 amino-acid long acidic model pro-peptide. The antimicrobial peptide is a fragment of the crotalicidin peptide, a member of the cathelidin family, from rattlesnake venom. The model pro-peptide is a deca (glutamic acid). Attachment of the model pro-peptide only leads to a moderately large reduction in the binding to- and induced leakage of model liposomes, while the antimicrobial activity of the crotalicidin fragment is completely inhibited by attaching the model pro-peptide. Attaching the pro-peptide induces a conformational change to a more helical conformation, while there are no signs of intra- or intermolecular peptide complexation. We conclude that inhibition of antimicrobial activity by the model pro-peptide might be related to a conformational change induced by the pro-peptide domain, and that additional effects beyond induced changes in membrane activity must also be involved.

## Introduction

Many proteins are synthetized as zymogens, precursor proteins that are inactive due to the presence of pro-peptide domains^1^. This also holds for antimicrobial peptides and proteins such as mammalian defensins^2^, cathelicidins^3^ and RegIII family proteins^4,5^. These are often synthesized and stored in inactive form, to protect host cells from potential cytotoxic effects as well as protecting the peptides and proteins and protected them from enzymatic degradation^6–8^. Understanding how pro-peptides control the activity of antimicrobial peptides is not only important in biology, but also for applications of antimicrobial peptides as pharmaceutical compounds. Biotechnologists have exploited fusion of antimicrobial peptides with pro-peptides to allow for the efficient production of antimicrobial peptides in bacterial hosts^9^. Pro-peptides can also be part of a pro-drug strategy for antimicrobial peptides, for example by arranging pro-peptide removal to be induced by the presence of a specific bacterial pathogen^10^.

Explicit demonstration that pro-peptides inactivate the biological activity of antimicrobial peptides has been given for a number of cases, such as for defensins^6–8^ but molecular mechanisms for inactivation have not yet been investigated. Very often, pro-peptide domains of antimicrobial peptides are highly acidic, and have charge densities that precisely neutralize the positive charge of the antimicrobial peptides. This suggests that electrostatic neutralization somehow plays an important role in inactivation by pro-peptides, but is unclear in what way. Ganz and co-workers^6,7^ go one step further by hypothesizing that (at least for defensins) inactivation is due to intramolecular interaction of the pro-domain with the antimicrobial peptide domain, but again, no structures have been presented yet from which this is apparent.

Inactivation of antimicrobial and cytotoxic activity are by no means the only possible functions of pro-domains. For example, Ganz and co-workers^11^ have shown that the acidic pro-peptide is necessary for the precise sub-cellular trafficking and sorting of proHNP1. The role of pro-peptide domains for cathelicidins is less clear than for the defensins. Cathelicidins^3,12,13^ are characterized by their highly conserved cathelin-like domain (CLD), a small folded domain with cystatin-like fold^14^, which is followed by a highly variable domain containing the antimicrobial peptide. One group has reported that the full-length precursor protein for the sole human cathelicidin, LL-37 (known as hCAP18) has no antimicrobial activity^15^, but a more recent report suggests that the antimicrobial activity of hCAP18 is practically identical to that of LL-37^16^. Hence, at this stage it is not yet clear whether for LL-37, its pro-sequence acts as an inhibitor of its antimicrobial activity or not.

Given the importance of acidic pro-peptides in controlling the activity of antimicrobial activity both in biology and in possible pharmaceutical applications of antimicrobial peptides, we here study the impact of acidic pro-peptides on the secondary structure, membrane interactions and antimicrobial activity in a simple model system. As an antimicrobial peptide, we use a fragment of crotalicidin (Ctn), an antimicrobial peptide from the venom of the rattlesnake (*Crotalus durissus*). Falcao *et al*.^17^ have studied the two fragments Ctn[1–14] and Ctn[15–34] that would be theoretically expected upon digestion with elastase. It was found that the short fragment Ctn[15–34] of full-length crotalicidin has a high antimicrobial activity, whereas the fragment Ctn[1–14] has no antimicrobial activity even though it is polycationic. The Ctn[15–34] peptide was also found to have a lower toxicity than full-length Ctn for human cells and was therefore suggested to be a potentially interesting antimicrobial peptide for further development as a pharmaceutical compound. Recently the mechanisms of membrane permeabilization for both model liposomes and bacterial membranes by Ctn and Ctn[15–34] were characterized in detail by Pérez-Peinado *et al*.^18^.

In the crotalicidin precursor protein, at the N-terminal side, the antimicrobially active Ctn peptide is preceded by a pro-domain, which is a highly negatively charged stretch of amino acids, rich in glutamic acids. The negative charge (24 negatively charged residues, 6 positively charged residues) approximately compensates the positive charge (15 positively charged residues) of the antimicrobial peptide. We here study the effect of N-terminally attaching a simple deca (glutamic acid) pro-piece (E_10_) to the crotalicidin fragment Ctn[15–34].

While neither the Ctn[15–34] peptide nor the E_10_-Ctn[15–34] peptide occurs naturally, we believe that these peptides can be a simple model for how pro-peptides modulate the physical and biological properties of much longer natural antimicrobial peptides. Also, the Ctn[15–34] peptide has favorable activities to be further developed for therapeutic applications^17^ and it is interesting to study how to different types of model pro-peptides modulate its activity, both for pro-drug strategies^10^, and for the biosynthetic production of such peptides^9^.

We find that attaching the E_10_ model pro-peptide leads to a significant change in the secondary structure of the peptide in solution and to a full inactivation of its antimicrobial activity. Surprisingly however, physical properties typically associated with antimicrobial activity, viz. adsorption to lipid bilayers and the induction of liposome leakage are only moderately affected.

## Material and Methods

### Materials

Ctn[15–34] (KKRLKKIFKKPMVIGVTIPF-NH_2_), E_10_-Ctn[15–34] and (GS)_4_-Ctn[15–34] were synthesized by Pepscan (Lelystad, the Netherlands). Ion-exchange chromatography was used to replace all counter ions by Na^+^. The peptides have a reported purity of more than 94%. Molecular masses for all peptides (2371.2 Da for Ctn[15–34] 3662.3 Da for E_10_-Ctn[15–34] and 2947.8 Da for (GS)_4_-Ctn[15–34]) were confirmed using mass-spectrometry by Pepscan. The full-length crotalicidin peptide (Ctn) was obtained from *Aminotech* Ltda (São Paulo, Brazil) (>95% of purity). The molecular mass of this peptide was also confirmed using mass-spectrometry. Concentrations of peptides in solution were determined using UV-spectroscopy. Micro centrifuge filters with a cut-off of 300 kDa were obtained from Pall Life Sciences Benelux (Mijdrecht, the Netherlands). The lipids 1,2-dioleoyl-sn-glycero-3-phosphocholine (DOPC) and 1,2-dioleoyl-sn-glycero-3-phospho-(1′-rac-glycerol) (DOPG) were purchased from Avanti Lipids (Alabaster, AL, USA). The fluorescent dye calcein was purchased in powder-form from Sigma-Aldrich. All other chemicals were of analytical grade, and were obtained from Sigma-Aldrich.

### Dynamic light scattering

Dynamic light scattering (DLS) was performed using a Malvern Nano ZS equipped with a 10 mW He-Ne laser with a wavelength of 632.4 nm. Measurements were done at a fixed scattering angle of 173°. Peptide solutions were filtered to remove dust particles and possible peptide aggregates that might interfere with the DLS measurements using 300 kDa cut-off micro centrifuge filters. For each sample, 20 μL of filtered peptide solution was transferred into a clean low-volume quartz cuvette. For each sample, 3 size measurements where done, with run times and number of run repetitions being determined by the Malvern DTS software version 7.12. In all cases, a distribution analysis performed using the Malvern DTS Software showed a single dominant diffusion peak. Hydrodynamic radii reported correspond to this dominant peak, standard deviations are for the 3 replicates of the size measurements.

### Circular Dichroism

Circular dichroism (CD) spectra were obtained using a Jasco-715 spectro-polarimeter equipped with a Peltier element for temperature control. Spectra were obtained in the far-UV range, from 190 to 260 nm. Spectra reported have been obtained by averaging 20 single scans and were obtained at a peptide concentration of 0.2 mg.mL^−1^, in a 10 mM K_2_HPO_4_ 50 mM Na_2_SO_4_ buffer pH 7.4 at 25 °C. For a quantitative interpretation of the spectra in terms of a percentage of α-helical structure, spectra were fitted using the CONTIN algorithm as implemented in the DICHROWEB^19,20^ webserver, using basis set #7.

### *In vitro* antimicrobial assays

For the *in vitro* antimicrobial assays, strains of *Eschericia coli* ATCC 25922, *Klebsiella pneumoniae* ATCC 13883 and *Staphylococcus aureus* ATCC 25923 were used. Additionally, we tested clinical isolates of *Klebsiella pneumoniae* carbapenemase (KPC) positive strains of *E*. *coli* (KPC + 001812446) and *K*. *pneumoniae* (KPC + 001825971). All strains were grown overnight in Mueller Hinton (MH) broth at 37 °C. Minimum inhibitory concentration (MIC) measurements were performed using 1 × 10^6^ CFU per mL^−1^ and serial 2-fold dilution series of the peptides Ctn[15–34] E_10_-Ctn[15–34] and (GS)_4_-Ctn[15–34] starting at 128 µg.mL^−1^. Following established protocols^21–23^ the MIC was determined after 24 h of incubation at 37 °C, and was taken to be the lowest peptide concentration with total inhibition of bacterial growth, as assessed by absorbance measurements in a polystyrene 96 well plates at 600 nm using a microplate reader (Bio-Tek PowerWave HT, EUA). Bacteria in MH broth containing the antibiotics (chloramphenicol, gentamicin and imipenem at 1 mg.mL^−1^) were used as negative and positive control respectively. Minimal bactericidal concentrations (MBCs) were determined by plating out 10 µl of the content on MH agar plates of the wells where no bacterial growth was observed. MBC was recorded as the lowest concentration at which no colonies were observed after 24 h incubation at 37 °C. In each case, three technical and biological replicates were used.

### Neutral-red (NR) *in vitro* toxicity assay with Caco-2 cells

Viability of Caco-2 cells at increasing peptide concentrations was determined using a Neutral-red (NR) assay as described previously^24^. After overnight incubation of the cells with the peptides Ctn[15–34] E_10_-Ctn[15–34] and (GS)_4_-Ctn[15–34] at peptide concentrations from 2–128 μg.mL^−1^, 10 μL of a 33 μg.mL^−1^ NR solution was added to the wells containing the peptide-incubated cells. After 3 more hours of incubation, the supernatant was removed and the cells were washed with PBS. Next, 150 μl of 1% acetic acid-50% ethanol was added and shaken for 10 min at room temperature. Finally, the absorbance was measured at 540 nm and 690 nm (background absorbance) using a SpectraMax M5 microplate reader (Molecular Devices). Readings were expressed as NR uptake relative to the uptake of the cells exposed to the negative control (medium or DMSO).

### Galleria mellonella *in vivo* toxicity assay

An *in vivo* toxicity assay was performed using *Galleria mellonella*^25^. *G*. *mellonella* larvae in their final instar stage were purchased (UK Waxworms Ltd, Sheffield, UK), and stored in the dark at 15 °C, and used within 14 days. Larvae were separated by weight, and only larvae between 0.2 and 0.3 g were used for experiments. Larvae were injected with 20 μL of peptide solutions, or controls in the left posterior proleg using Terumo Myjector 29 G insulin syringes (VWR International). The syringes were changed between different treatments. Two negative control groups were included in every experiment; one group was not injected to control for background larval mortality (no manipulation control) and the other group (uninfected control) was injected with PBS to control for the possible effect of physical trauma on mortality. After injection, larvae were stored in Petri dishes in the dark at 37 °C with 5% CO_2_ for up to 144 h post-infection (p.i.) and inspected every 24 h for survival; larvae were considered dead if they did not move after shaking of the petri dish. For each sample (non-manipulated control, water control, peptides) fifteen randomly chosen larvae were used. The peptide concentration was 10 mg per kg of body weight.

### Liposome leakage assay

Following earlier work^26^, lipids DOPC and DOPG were dissolved in chloroform at a concentration of 25 mg.mL^−1^, mixed in a molar ratio of 7:3, and subsequently diluted further to 10 mg.mL^−1^ using chloroform. The chloroform was evaporated using rotary evaporation (350 mbar, 313 K, 100 rpm). The lipid film was then dried in vacuum for at least 2 h after which the lipids were re-suspended in calcein-containing buffer (70 mM calcein in 10 mM Tris-HCl, pH 7.5) to get a 30 mM lipid suspension by hydration for 1 h in a rotary evaporator (no vacuum, 323 K, 100 rpm). Multilamellar vesicles thus formed were freeze-thawed four times using liquid nitrogen and a 310 K water bath to get unilamellar vesicles. A mini-extruder (Avanti Lipids) equipped with a 200 nm pore size polycarbonate membrane was used to perform 21 extrusions to homogenize the size of the lipid vesicles. Vesicles were separated from free calcein on a gravity driven Sephadex G-50 size exclusion column and eluted with a 10 mM Tris-HCl buffer containing 100 mM NaCl (pH = 7.5). The vesicles were characterized using dynamic light scattering, for which an ALV instrument equipped with an ALV5000/60 × 0 external correlator and a 300 mW Cobolt Samba-300 DPSS laser operating at a wavelength (λ) of 532 nm was applied. A cumulant analysis showed the vesicles to be monodisperse, and to have an average diameter of 190 ± 5 nm. The degree of dilution of the vesicles during size exclusion chromatography was estimated by comparing the intensity of scattered light (count rate) before and after size-exclusion chromatography. For this scattering angles θ ranging from 20° to 140° were used with steps of 5°, five measurements of 30 s were recorded for each scattering angle and the average was taken over all measurements. A fluorescence filter was applied that only transmits the laser light, to prevent the fluorescence light emitted by calcein from disturbing the measurements. Vesicles used for the leakage assay were diluted with a 10 mM Tris-HCl buffer containing 100 mM NaCl (pH = 7.5) to a final lipid concentration of 50 μM. Peptides were dissolved in the same buffer, at a concentration of 6.4 mg.mL^−1^. The calcein leakage caused by the AMPs was measured by following the fluorescence intensity of the liposome solutions over time in a Cary Eclipse cuvette fluorimeter using an excitation wavelength of 490 nm and an emission wavelength of 520 nm. The widths of the excitation and emission slits were 2.5 nm. The fluorescence measurements were started with 588 μL of the vesicle solution without AMPs. After two minutes the fluorescence intensity signal was stable and 12 μL of 6.4 mg.mL^−1^ AMP solution was added, to achieve a final peptide concentration of 128 μg.mL^−1^, equal to the highest peptide concentration used in the MIC assays. In total, the measurements lasted 10 minutes. At the end of the experiments, 10 μL of a 10% (v/v) Triton X-100 solution was added, which is a surfactant that disrupts the liposomes. The signal after Triton addition was taken to correspond to 100% liposome leakage. For all peptides, the liposome leakage assays was repeated four times with freshly prepared liposomes and results were found to be reproducible.

### Reflectometry of peptides adsorbing on supported lipid bilayers

Adsorption measurements were performed using an optical reflectometer with a stagnation-point flow cell and an oxidized silicon surface as a substrate, as described by Dijt *et al*.^27^. The laser beam reflects from the surface at exactly the point where the jet flow reaches the surface. The reflectometer signal can be directly converted into an adsorbed amount (Γ) if the refractive index increment of the adsorbing species is known^27,28^. The refractive index increment for lipids is d*n*/d*c* ≈ 0.145 μg-mL^−1^ ^29,30^ that of proteins is d*n*/d*c* ≈ 0.185 μg-mL^−1^ ^31^ Clean oxidized silicon strips (with a silica layer of ~62 nm) were prepared as follows. Silica strips were washed with demiwater, sonicated for 1 min in 2-propanol, washed in milliQ water, dried under a nitrogen stream and plasma cleaned for 2 min before being stored in milliQ water. The strips were used in the experiment within 5 h of cleaning. A supported lipid bilayer (SLB) was formed on top of the silica surface by means of vesicle adsorption and subsequent rupture and SLB formation at high surface coverage^32^. Vesicles were prepared as described for the liposome leakage assay, except for the fact that no calcein was used. For preparing the SLB, vesicles were diluted in 10 mM Tris, 100 mM NaCl, pH 4 to a final concentration of 200 μM of lipids. To promote adsorption of the negatively charged vesicles, the silica substrates were first equilibrated against a low pH buffer (10 mM Tris, 100 mM NaCl, pH 4). At this low pH, the silica is less negatively charged as opposed to neutral pH. After a steady baseline was obtained, the flow was switched to the 200 μM vesicle solution for a few minutes, until the signal stabilized again. Next, the flow was switched back to the low pH buffer and rinsed for some minutes until the signal stabilized again and finally, flow was switch to a neutral pH buffer (10 mM Tris, 100 mM NaCl, pH 7.5). This finalized the procedure to attach the SLB. Next, flow was switched to a solution of peptides at a concentration of 16 μg-mL^−1^ in 10 mM Tris 100 mM NaCl, pH 7.5. Reproducibility of these measurements was typically found to be around 10%.

### Molecular Modelling

First, Blastp^33^ was used to find the best template structure for the molecular modeling of the solution structure of the peptides Ctn[15–34] E_10_-Ctn[15–34] and (GS)_4_-Ctn[15–34]. The solution nuclear magnetic resonance (NMR) structure of crotalicidin in DPC micelles (PDB entry: 2MWT) isolated from the Rattlesnake (*Crotalus durissus*) venom^17^ was selected for the construction of a set of 100 tridimensional theoretical models using Modeller v. 9.17^34^. For each of the three peptides, the lowest free-energy theoretical model from the set was then selected and used for validation procedures: PROCHECK^35^ was used to evaluate peptide geometry, stereochemistry and energy distributions, as well for calculating average scores (G-factor) for dihedral angles and bond lengths. ProSA-web^36^ was used to evaluate the fold quality of the selected models.

### Molecular dynamics

Molecular dynamics simulations for Ctn[15–34] E_10_-Ctn[15–34] and (GS)_4_-Ctn[15–34] were carried out in explicit water, using the single point charge (SPC) water model. All simulations were performed using the GROMOS96 43a1 force field from the computational package GROMACS v.5.0.4^37^. The validated tridimensional theoretical models for the peptides obtained as described above were used as initial structures in all simulations. Chloride and sodium ions were also added to neutralize the system’s charge. The geometry of water and molecules was constrained using the SETTLE algorithm^38^. Moreover, the LINCS algorithm was used to link all the atom bond length. Particle Mesh Ewald (PME) was also used for electrostatic corrections, with a radius cut-off of 1.4 nm to minimize the computational simulation time. The same radius cut off was also used for van der Waals interactions. The list of neighbours of each atom was updated every 10 simulation steps of 2 fs each. Before the start of the MD simulations, a steepest descent algorithm (50,000 steps) was applied for energy minimization. After that, the system underwent a normalization of temperature and pressure to 300 K and 1 bar, using the velocity rescaling thermostat (NVT) and the Parrinello-Rahman barostat (NPT), respectively, for 100 ps. The system with minimized energy and balanced temperature and pressure was submitted to molecular dynamics simulation during 100 ns, in triplicate.

## Results

### Peptide sequences

**Figure 1:**
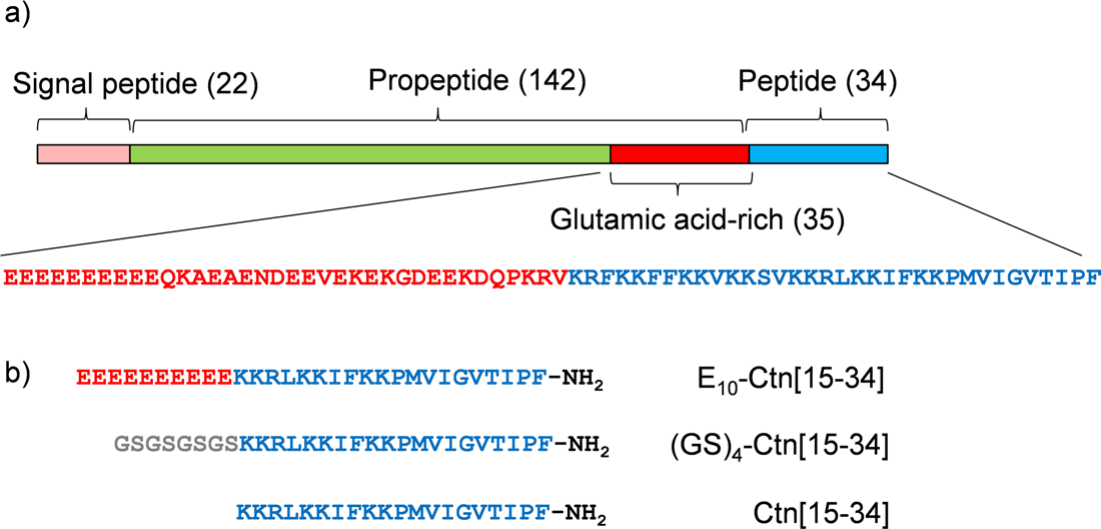
Crotalicidin-derived peptides used in this study. (**a**) Domain structure of crotalicidin pre-pro-peptide (Uniprot U5KJM4) in brackets: length of domains in numbers of amino acids. Flanking the peptide, the pro-peptide features a 35 amino acids long glutamic acid rich domain, whose sequence is also given in red (21 negatively charged residues, 6 positively charged residues), together with that of the full-length crotalicidin peptide in blue (15 positively charged residues). (**b**) Short model peptides inspired by full-length crotalicidin peptide and pro-peptide, used in this study. The Ctn[15–34] fragment has 7 positively charged residues.

The domain structure of the Ctn precursor protein is shown in Fig. 1a. On the N-terminal side of the Ctn peptide, the Ctn precursor protein has a pro-peptide that is 142 residues long. The C-terminal 35 residues of the pro-peptide, that connect to the Ctn peptide, is extremely rich in glutamic acid residues and has a very high net negative charge (21 negatively charged residues and 6 positively charged residues). The net negative charge of this part of the pro-peptide approximately compensates for the net positive charge of full length Ctn (15 positively charged residues, no negatively charged residues). Pro-peptides with long oligo (glutamic acid) stretches are found for many other AMPs^9^. As a model pro-peptide for the short but highly antimicrobially active Ctn[15–34] fragment, mimicking the glutamic acid-rich domain of the crotalicidin pro-peptide, we here choose a simple deca(glutamic acid), E_10_. As a control for the effect of losing the free N-terminal on the activity of Ctn[15–34] we use a glycine/serine sequence: such sequences are often used in fusion proteins as inert, hydrophilic flexible linkers. Additionally and important for the case of AMPs, the glycine and serines have uncharged side chain, and therefore do not change the net AMP charge. Thus we arrived at the three peptide sequences (Fig. 1b) that we study here: Ctn[15–34] E_10_- Ctn[15–34] and (GS)_4_-Ctn[15–34]. For N-terminal glutamic acid residues, pyroglutamization may be a concern, therefore we performed MALDI-TOF MS and MS/MS to demonstrate the absence of pyroglutamization of the N-terminal glutamic acid for the E_10_- Ctn[15–34] peptide (Supplementary Information Figs SI–1–2).

### Characterization of peptides in solution

We first explored the solubility and aggregation state of the peptides, using dynamic light scattering (DLS), in both a low and high ionic strength buffer, at neutral pH. Results are shown in Table 1. All peptides dissolved well and did not appear to aggregate or self-assemble in solution, even at high concentrations employed for the DLS experiment (10 mg.mL^−1^). There is a clear difference between the small solution sizes of Ctn[15–34] (2.2 ± 0.1 nm) and (GS)_4_-Ctn[15–34] (2.4 ± 0.1 nm), and the larger solution sizes of E_10_-Ctn[15–34] (4.0 ± 0.1 nm). Also, the solution sizes of Ctn[15–34] and (GS)_4_-Ctn[15–34] increase by 45% and 33% respectively with ionic strength, whereas that of E_10_-Ctn[15–34] remains constant. Although the sizes most likely reflect the solution sizes of individual peptides, we cannot completely exclude that a small degree of self-association contributes to the measured sizes. Being a block-polyampholyte, at low ionic strength, one might have expected the E_10_-Ctn[15–34] to aggregate or coacervate as a consequence of electrostatic interactions, but this was not observed: this should have resulted in much larger solution sizes as measured in DLS. Presumably, the lengths of the charged blocks were still too short, or the charged blocks were too asymmetric in (in terms of numbers charges) for coacervation to occur. Instead, we observe that at 100 mM of added NaCl the E_10_-Ctn[15–34] the peptide is no longer fully soluble at 10 mg.mL^−1^.

**Table 1:** Hydrodynamic diameter as determined using dynamic light scattering of 10 mg.mL^−1^ of the peptides in the indicated buffers: PB (10 mM Pi pH 7.4) and PBS (10 mM Pi pH 7.4 + 100 mM NaCl).

Buffer	10 mM Pi pH 7.4	10 mM Pi pH 7.4 + 100 mM NaCl
	*D*_*H*_ (nm)	*D*_*H*_ (nm)
Ctn[15–34]	2.2 ± 0.1	3.2 ± 0.1
(GS)_5_-Ctn[15–34]	2.4 ± 0.1	3.2 ± 0.1
E_10_-Ctn[15–34]	4.0 ± 0.3	4.1 ± 0.3

For 10 mg.mL^−1^ E_10_-Ctn[15–34] in PBS we observed phase separation, therefore the experiment was done at the lower concentration of 2 mg.mL^−1^ where the system was in a one-phase region.

**Figure 2:**
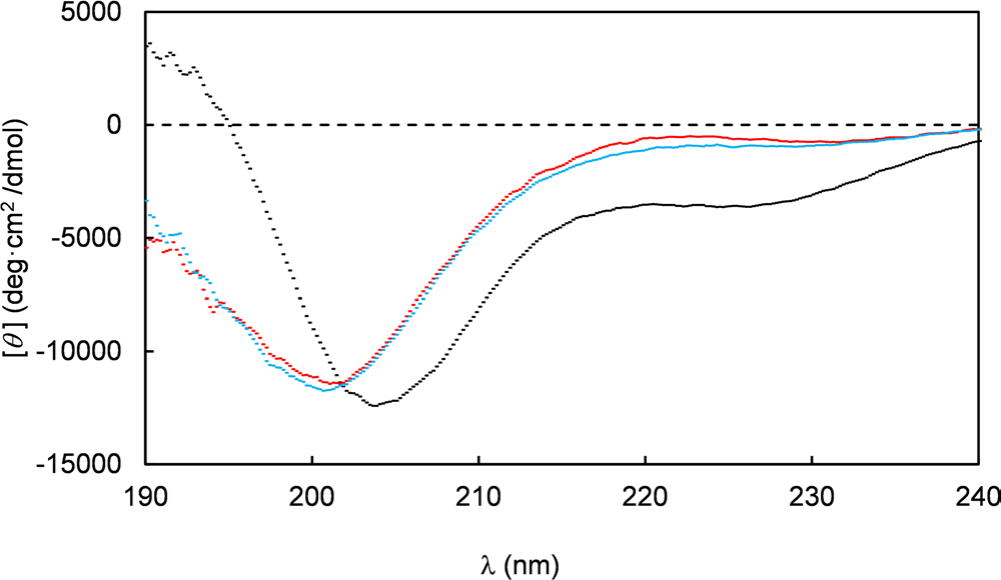
Circular dichroism spectra of the peptides Ctn[15–34] (red), E_10_-Ctn[15–34] (black) and (GS)_4_-Ctn[15–34] (blue). Residue molar ellipticity [θ] in deg.cm^2^/dmol is plotted versus the wavelength (λ in nm). Measurements were performed at a peptide concentration of 0.2 μg.mL^−1^ in a 10 mM K_2_HPO_4_ 50 mM Na_2_SO_4_ buffer, pH 7.4.

The larger solution size of the E_10_-Ctn[15–34] peptide possibly reflects a different secondary structure, therefore we next performed circular dichroism (CD) measurements for each of the peptides. Spectra were acquired for 2 μg-mL^−1^ peptide solutions in a 10 mM potassium phosphate buffer at pH 7.4 (Fig. 2). We find that the E_10_-Ctn[15–34] spectrum is notably different from that of (GS)_4_-Ctn[15–34] and Ctn[15–34] which essentially overlap with each other. A qualitative comparison with typical CD spectra for random coil versus α-helical configurations shows that the (GS)_4_-Ctn[15–34] and Ctn[15–34] spectra are characteristic of predominantly random coil configurations, whereas that of E_10_-Ctn[15–34] is indicative for a substantial contribution from α-helices. For a more quantitative assessment we have used a CONTIN algorithm to fit the CD spectrum and extract percentages of α-helical structure. The estimated percentages of α-helical structure are 21% for E_10_-Ctn[15–34] and, respectively, 3% and 4% for Ctn[15–34] and (GS)_4_-Ctn[15–34]. This confirms the qualitative interpretation that the deca(glutamic acid) pro-peptide significantly increases the overall α-helicity of the Ctn[15–34] whereas the addition of the flexible hydrophilic (GS)_4_ sequence does not. The increased helicity of E_10_-Ctn[15–34] could be related to the well-known propensity of glutamic acid residues to stabilize α-helices. Similarly, the more unfolded structure of (GS)_4_-Ctn[15–34] could be related propensity of the glycines to form loops and destabilize α-helices, as previously demonstrated by Shingate and Showdhani^39^. A more helical structure for E_10_-Ctn[15–34] also matches well with the observation of larger solution sizes by DLS for this peptide.

**Figure 3:**
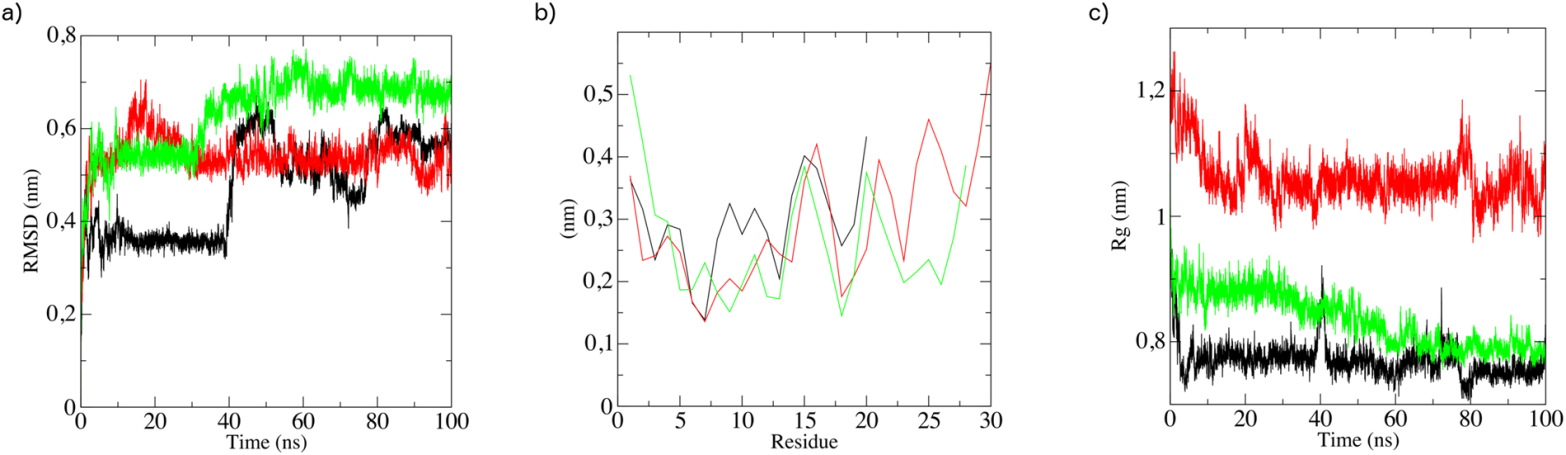
Molecular dynamics simulations in water (100 ns) for the peptides Ctn[15–34] (black curves), E_10_-Ctn[15–34] (red curves) and (GS)_4_-Ctn[15–34] (green curves), yielding the parameters (**a**) Root mean square deviation (RMSD) of single run, (**b**) root mean square fluctuations (RMSF) and (**c**) radius of gyration (*R*_*g*_). Results are for single runs, three runs were done, for each peptide, with similar results.

**Figure 4:**
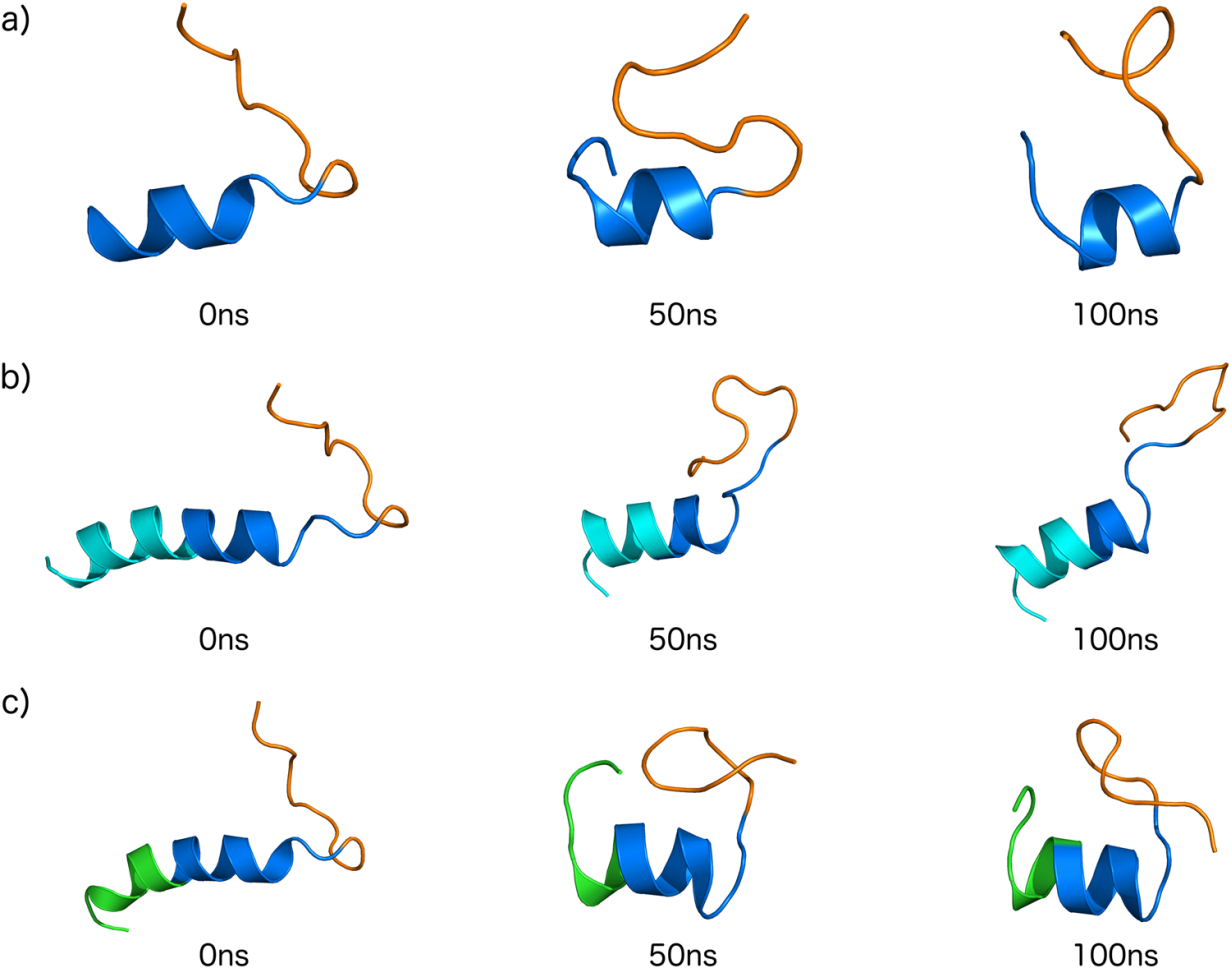
Theoretical structures snapshots of (**a**) Ctn[15–34] (**b**) E_10_-Ctn[15–34] and (**c**) (GS)_4_-Ctn[15–34] during 100 ns of dynamics simulations (50 ns intervals). Domain colors: basic domain Ctn[15–34] – dark blue, hydrophobic domain Ctn[15–34] - orange, acidic E_10_ domain – light blue, control pro-peptide domain (GS)_4_ – green.

For an indication as to where helicity could be induced by the E_10_ model pro-peptide we have performed molecular dynamics (MD) simulations, starting from initial theoretical structures generated by molecular modelling studies (Supplementary Information Fig. SI-3 and Table SI−1) and which are based on the solution NMR structure of full-length crotalicidin in DPC micelles^17^. The micellar environment typically induces a high α-helical content of antimicrobial peptides, so it may be expected that the MD runs for the peptides in water show at least partial unfolding. Indeed, during the 100 ns runs, all peptides develop large root mean square deviations (RMSD) as compared to the initial (Fig. 3a). A root mean square fluctuation (RMSF) analysis (Fig. 3b) for the equilibrated configurations indicates that for Ctn[15–34] and E_10_-Ctn[15–34] it is especially the C-terminus that exhibits the largest flexibility, whereas for (GS)_4_-Ctn[15–34] the largest flexibility is exhibited by the N-terminal residues. For all peptides, the loss of α-helicity is accompanied by a decrease of ~0.2 nm in their radius of gyration (*R*_g_) (Fig. 3c). Representative snapshots of conformations of the peptides at simulation times of 0, 50 and 100 ns are shown in Fig. 4.

Trends observed in the MD simulations agree with the experimental observations using DLS and CD: the E_10_-Ctn[15–34] peptide has a larger gyration radius and larger α-helicity, as compared to both the Ctn[15–34] and the (GS)_4_-Ctn[15–34] peptides. The current level of simulations however is not yet good enough to quantitatively compare experimental to theoretical α-helicities. For example, from the CD we find an almost no α-helicity for the (GS)_4_-Ctn[15–34] peptide in solution (4%), but the simulation snapshots show that significant α-helicity remains after 100 ns (Fig. 4c). Taking the simulations therefore as a qualitative indication for structural changes induced by attaching the acidic E_10_ and the control (GS)_4_ “pro-peptides” to Ctn[15–34] we conclude that the hydrophobic C-terminal part Ctn[15–34] (colored orange in Fig. 4) remains largely unstructured irrespective of the attachments at the N-terminal side. When attaching E_10_, it is the E_10_ peptide itself that presumably contributes mostly to the additional helicity that we observe in CD, and hence also to the increase in the solution size observed in DLS.

### Antimicrobial activity and toxicity

All three peptides were tested against different bacterial strains, both Gram negative and Gram positive, in order to determine their *in vitro* antibacterial activity: *E*. *coli* ATCC 25922, *K*. *pneumoniae* ATCC 13883 and *S*. *aureus* ATCC 25923. Additionally, we considered Gram-negative clinical isolates from KPC positive *E*. *coli* (KPC + 001812446) and *K*. *pneumoniae* (KPC + 001825971) due the medical relevance related to KPC strains by their resistance to conventional antibiotics. MIC and MBC results are given in Table 2.

**Table 2:** MIC and MBC for the peptides Ctn[15–34] E_10_-Ctn[15–34] and (GS)_4_-Ctn[15–34] against Gram-negative and -positive bacteria.

Microorganism	Gram	MIC*(MBC) μg.mL^−1^	E_10_-Ctn[15–34]	(GS)_4_-Ctn[15–34]
		Ctn[15–34]		
*E*.*coli* ATCC 25922	−	2(2)	>128 (>128)	2(2)
*E*.*coli* (KPC + 001812446)	−	2(2)	>128 (>128)	2(2)
*K*. *pneumoniae* ATCC 13883	−	2(2)	>128 (>128)	2(16)
*K*. *pneumoniae* (KPC + 001825971)	−	8(8)	>128 (>128)	16(32)
*S*. *aureus* ATCC 25923	+	16(>128)	>128 (>128)	64(>128)

MIC and MBC determinations were carried out in triplicate, with no difference in outcome.

Results for Ctn[15–34] confirm those previously obtained by Falcao^17^: the peptide is very active against Gram-negative bacteria presenting all the MIC values less than 8 μg.mL^−1^, and somewhat less against Gram-positive *S*. *aureus*. Additionally, We find that Ctn[15–34] is also active against a clinical isolate (see Table 2). In stark contast, for E_10_-Ctn[15–34] we find an absence of any antimicrobial activity up to 128 μg.mL^−1^. Hence the E_10_ sequence is indeed a very good model for an inhibitory pro-peptide. Inhibition of activity is not due to the absence of a free N-terminus: the activity of the control peptide (GS)_4_-Ctn[15–34] is identical to that of Ctn[15–34] except for *K*. *pneumoniae* KPC and *S*. *aureus*, in which cases the MIC values are 16 and 64 μg.mL^−1^, respectively.

**Figure 5:**
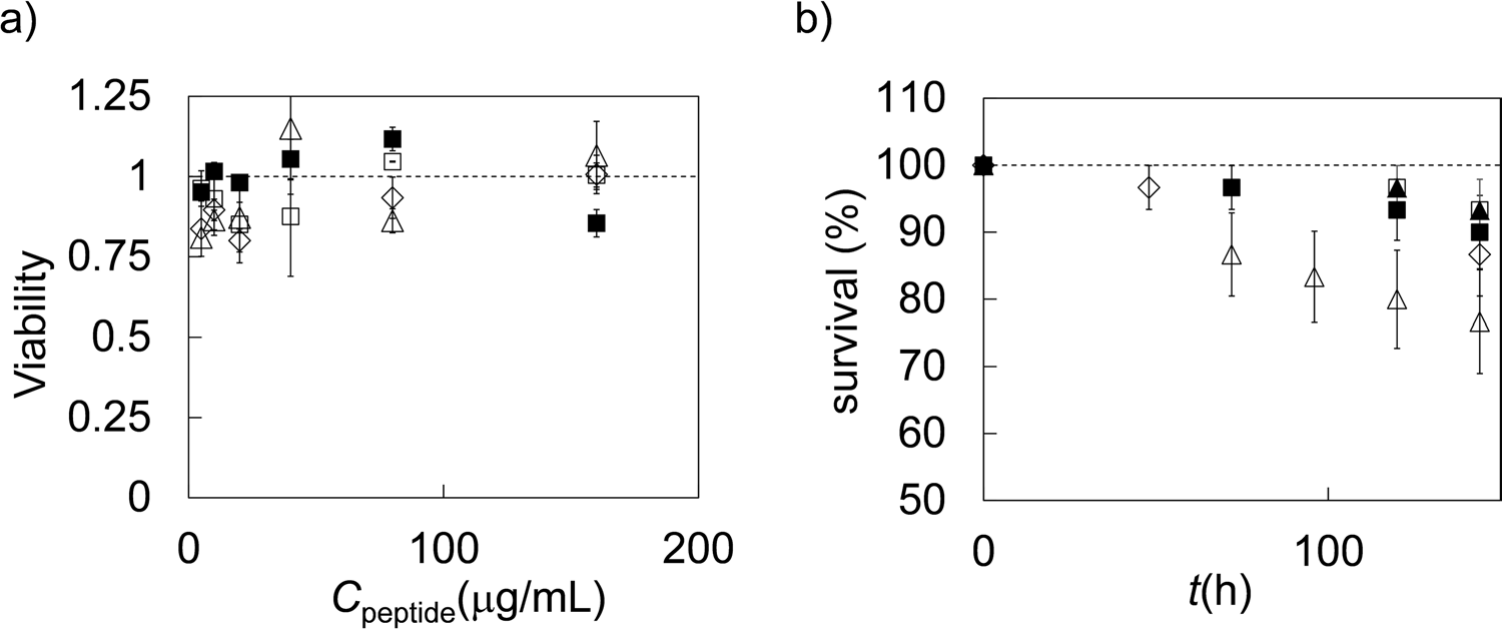
Toxicity of peptides Ctn[15–34] E_10_-Ctn[15–34] and GS_4_-Ctn[15–34]. (**a**) *In vitro* neutral red uptake assay for Caco-2 cells. Viability (fraction of viable cells) as compared to untreated Caco-2 cells as a function of peptide concentration. Diamonds: filled squares: PBS control, open squares: Ctn[15–34] open diamonds: E_10_-Ctn[15–34] open triangles: (GS)_4_-Ctn[15–34]. Error bars are standard deviations of replicate measurements. (**b**) *In vivo* toxicity of the peptides for *Galleria mellonella* larvae. Percentage surviving larvae as a function of time. Filled squares: H_2_O control, filled triangles: no treatment control, open triangles: Ctn[15–34] open diamonds: E_10_-Ctn[15–34] open squares: (GS)_4_-Ctn[15–34]. For each peptide a group of n = 15 larvae were used, error bars are standard deviations within the group.

We have also studied the effect of the attachment of the pro-peptide on the toxicity of the peptides to eukaryotic cells *in vitro*, and on the toxicity of the peptides *in vivo* using *Galleria mellonella* larvae. A neutral red uptake (NRU) assay was used to access viability of the mammalian cell line Caco-2. Monolayers of this cell mimic the human intestinal epithelium and are widely used as a model of human intestinal absorption. Results are shown in Fig. 5a. The NRU assay indicates complete cell viability after overnight incubation over the entire concentration range studied for all the peptides. To confirm this result a more sensitive test was used, the *in vivo* toxicity of the peptides for *G*. *mellonella* larvae. Following incubation with 10 mg.kg^−1^ of body weight of the peptides, survival of the larvae was measured up to 144 h (Fig. 5b). The Ctn[15–34] peptide is more toxic than the other peptides: larvae treated with this peptide have the lowest fraction of survival (Fig. 5b). Within the experimental margin of error, survival curves for the peptides E_10_-Ctn[15–34] and (GS)_4_-Ctn[15–34] were identical to those of the two controls, indicating that the addition of the (GS)_4_ sequence appears to reduce the *in vivo* toxicity but not the antimicrobial activity of Ctn[15–34].

### Interaction of the peptides with model membranes

The peptide physical-chemical properties that have been suggested to correlate most strongly with antimicrobial activity are their affinity towards model membranes, and their ability to induce leakage in model liposomes. Therefore, we have tested whether the E_10_ model pro-peptide prevents adsorption of the peptides on model membranes, and whether it prevents leakage from model liposomes. As a model for the predominantly negatively charged bacterial membranes, we use a 7:3 mixture (molar ratio) of the lipids DOPC and DOPG. Liposomes were prepared with an average diameter of 190 ± 5 nm, as determined using DLS.

**Figure 6:**
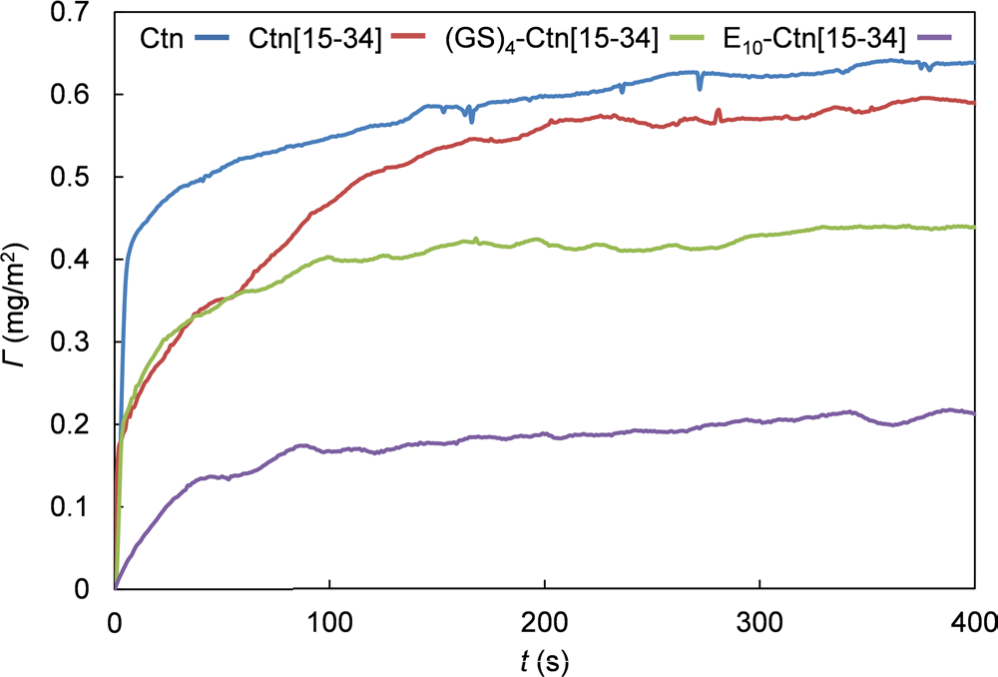
Adsorption of the peptides Ctn, Ctn[15–34] E_10_-Ctn[15–34] and GS_4_-Ctn[15–34] to mixed DOPC:DOPG (7:3 molar ratio) supported lipid bilayers (SLB). Peptide mass *Γ* (mg/m^2^) adsorbing to the SLB as a function of time. Curves from top to bottom are for Ctn, Ctn[15–34] GS_4_-Ctn[15–34] and E_10_-Ctn[15–34] Peptide concentrations are 16 μg.mL^−1^ in 10 mM Tris 100 mM NaCl, pH 7.5.

In a first experiment, supported lipid bilayers (SLB) were prepared on a silica wafer by adsorbing the liposomes to the silica at low pH. Subsequently, the amount of peptide (mg/m^2^) adsorbing to the SLB was monitored using reflectometry, as a function of time (Fig. 6). Note that in these experiments, we have also included the full-length crotalicidin peptide (Ctn). The reflectometry experiments demonstrated that, under the conditions of the experiment (10 mM Tris 100 mM NaCl, pH 7.5, and a peptide concentration of 16 μg.mL^−1^) both full length Ctn and the fragment Ctn[15–34] adsorb to the model membranes at the same surface density of approximately 0.5 mg.m^−2^. Attaching the (GS)_4_ sequence reduces the adsorbed amount to approximately 0.3 mg.m^−2^. Attaching the E_10_ model pro-peptide sequence leads to a further reduction of the adsorbed but the E_10_ model pro-peptide sequence by no means completely prevents adsorption: the adsorbed amount remains significant at approximately 0.15 mg.m^−2^.

**Figure 7:**
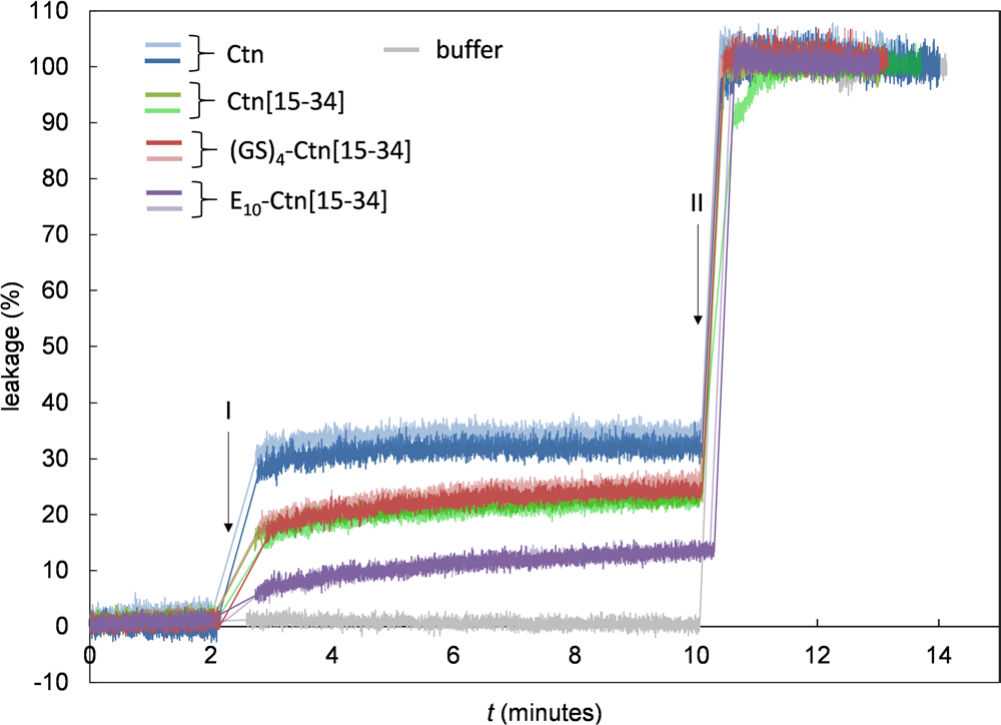
Liposome leakage assay for the peptides Ctn, Ctn[15–34] E_10_-Ctn[15–34] and (GS)_4_-Ctn[15–34]. Percentage of leakage as a function of time *t*(s). Mixed DOPC:DOPG (7:3 molar ratio) liposomes with a diameter of 190 nm filled with calcein (70 mM inside the liposomes), at a lipid concentration of 50 μM, were exposed to 128 μg.mL^−1^ of the peptides. Peptides were injected at *t* = 2 min (I). At *t* = 10 min (II) a small amount of 10% (v/v) Triton X-100 was added to release all of the calcein. Two replicates are shown for each peptide with different colors as indicated in the figure.

Next we measured liposome leakage induced by the various peptides. DOPC/DOPG liposomes filled with 70 mM of calcein (internal concentration) were exposed to 128 μg.mL^−1^of the peptides, and the fluorescence increase due to liposome leakage was monitored over time. Results for the percentage of liposome leakage induced by full length Ctn, Ctn[15–34] E_10_-Ctn[15–34] and (GS)_4_-Ctn[15–34] as a function of time are shown in Fig. 7. Leakage is greatest for the full length Ctn peptide (30% after 8 min.). The induced leakage for the Ctn[15–34] fragment and the (GS) _4_-Ctn[15–34] is the nearly the same (around 20% after 8 min.). Finally, the leakage induced by the E_10_-Ctn[15–34] peptide is the lowest of all peptides tested, but the E_10_ pro-peptide by no means completely prevents induced leakage (which is around 12% after 8 min.). Note that the absolute values of the induced leakage that we find for the Ctn and Ctn-derived peptides are similar to those found for other AMPs at similar conditions^40,41^.

Both experiments on model membranes therefore present results that do not correlate precisely with the observed antimicrobial activities: the addition of the (GS)_4_ leads to a significantly lower adsorption, but leakage and antimicrobial activity are not affected. Most strikingly, the addition of the E_10_ model pro-peptide leads to a complete inactivation of the antimicrobial activities, but only to a somewhat lower adsorption on model membranes, and to a only a somewhat lower induced leakage of model liposomes, in contrast to other studies^8^ where the presence of a pro-peptide was shown to lead not only to inhibition of antimicrobial activity, but also to a much lower adsorption on- and leakage of model liposomes.

## Concluding Remarks

We have found for a crotalicidin-derived antimicrobial peptide, Ctn[15–34] that the N-terminal addition of a deca(glutamic acid) model “pro-peptide” (E_10_) very effectively inhibits the antimicrobial activity. Earlier suggestions in the literature were that inhibition of antimicrobial activity is caused by charge neutralization and intra-molecular complexation of the antimicrobial peptide with its acidic pro-region^6,7^, leading to a reduction in membrane activity^8^. For the model system introduced here however, we find no indications for intra- or intermolecular (electrostatic) complexation at the solution conditions that we have tested here. Also, while there is complete inhibition of antimicrobial activity, there is only a moderate reduction in membrane binding and induced liposome leakage when N-terminally attaching the E_10_ model pro-peptide to the Ctn[15–34] antimicrobial peptide. The N-terminal attachment of the E_10_ model pro-peptide significantly increases the α-helicity of the peptide in solution, and for now we can only speculate that this conformational change might play a role in the inhibition of the antimicrobial activity.

In summary, our peptide model for the inhibition of antimicrobial activity by acidic pro-peptides partly calls into question mechanisms for the inhibition suggested earlier (charge neutralization, inter- and intramolecular complexation, leading to loss of membrane activity and hence loss of antimicrobial activity). We suggest that conformational changes induced by attaching the pro-peptides may be an additional factor determining the loss of antimicrobial activity.

For further progress in elucidating how acidic pro-peptides inhibit activity of antimicrobial peptides, it will be necessary to probe the activity and mechanism of various antimicrobial peptides with and without their pro-peptides in still more detail, for example by using a membrane potential assay^42^. Additionally, it will be neccesary to obtain more detailed structural information for complete precursor proteins and the corresponding antimicrobial peptides.

## Electronic supplementary material

supplementary information

## References

[1] Neurath H, Walsh K (1976). Role of proteolytic enzymes in biological regulation (A Review). A P. Natl. Acad. Sci. USA.

[2] Salzman NH (2010). Enteric defensins are essential regulators of intestinal microbial ecology. Nat. Immunol..

[3] Shinnar AE, Butler KL, Park HJ (2003). Cathelicidin family of antimicrobial peptides: proteolytic processing and protease resistance. Bioorg. Chem..

[4] van Ampting MTJ (2012). Intestinally secreted C-Type lectin Reg3b attenuates salmonellosis but not listeriosis in mice. Infect. Immun..

[5] Cash HL, Whitham CV, Behrendt CL, Hooper LV (2006). Symbiotic bacteria direct expression of an intestinal bactericidal lectin. Science.

[6] Michaelson D, Rayner J, Couto M, Ganz T (1992). Cationic defensins arise from charge neutraIized propeptides: a mechanism for avoiding leukocyte autocytotoxicity?. J. Leukocyte Biol..

[7] Valore EV, Martin E, Harwig SS, Ganz T (1996). Intramolecular inhibition of human defensin HNP-1 by its propiece. J. Clin. Invest..

[8] Satchell DP (2003). Interactions of mouse Paneth cell α-defensins and α-defensin precursors with membranes. J. Biol.Chem..

[9] Vassilevski AA, Kozlov SA, Grishin EV (2008). Antimicrobial peptide precursor structures suggest effective production strategies. Recent Pat. Inflamm. Allergy Drug Discov..

[10] Forde E, Devocelle M (2015). Pro-moieties of antimicrobial peptide prodrugs. Molecules.

[11] Liu L, Ganz T (1995). The pro region of human neutrophil defensin contains a motif that is essential for normal subcellular sorting. Blood.

[12] Zanetti M, Gennaro R, Scocchi M, Skerlavaj B (2000). Structure and biology of cathelicidins. Adv. Exp. Med. Biol..

[13] Tomasinsig L, Zanetti M (2005). The cathelicidins - structure, function and evolution. Curr. Opin. Prot. Pept. Sci..

[14] Yang Y, Sanchez JF, Strub MP, Brutscher B, Aumelas A (2003). NMR structure of the cathelin-like domain of the protegrin-3 precursor. Biochemistry-US.

[15] Zaiou M, Nizet V, Gallo RL (2003). Antimicrobial and protease inhibitory functions of the human cathelicidin (hCAP18/LL-37) prosequence. J. Invest. Dermatol..

[16] Pazgier M (2013). Structural and functional analysis of the pro-domain of human cathelicidin, LL-37. W. Biochemistry-US.

[17] Falcao CB (2015). Structural dissection of crotalicidin, a rattlesnake venom cathelicidin, retrieves a fragment with antimicrobial and antitumor activity. J. Med. Chem..

[18] Pérez-Peinado C (2018). Mechanisms of bacterial membrane permeabilization by crotalicidin (Ctn) and its fragment Ctn(15–34), antimicrobial peptides from rattlesnake venom. Biol. Chem..

[19] Whitmore L, Wallace BA (2008). Protein secondary structure analyses from circular dichroism spectroscopy: methods and reference databases. Biopolymers.

[20] Whitmore L, Wallace BA (2004). DICHROWEB, an online server for protein secondary Structure analyses from circular dichroism spectroscopic data. Nucleic Acids Research.

[21] *Performance Standards for Antimicrobial Susceptibility Testing Twenty-Second Informational Supplement*; 12nd ed. CLSI Supplement M100-S22 (Clinical and Laboratory Standards Institute, 2012)

[22] *Performance Standards for Antimicrobial Susceptibility Testing*. 27th ed. CLSI Supplement M100. (Clinical and Laboratory Standards Institute, 2017)

[23] Wiegand I, Hilpert K, Hancock RE (2008). Agar and broth dilution methods to determine the minimal inhibitory concentration (MIC) of antimicrobial substances. Nat. Protoc..

[24] Repetto G, del Peso A, Zurita JL (2008). Neutral red uptake assay for the estimation of cell viability/cytotoxicity. Nat. Protoc..

[25] Velikova N, Kavanagh K, Wells JM (2016). Evaluation of Galleria mellonella larvae for studying the virulence of Streptococcus suis. BMC Microbiol..

[26] Pera H, Nolte TM, Leermakers FAM, Kleijn JM (2014). Coverage and disruption of phospholipid membranes by oxide nanoparticles. Langmuir.

[27] Dijt JC, Stuart MAC, Hofman JE, Fleer GJ (1990). Kinetics of polymer adsorption in stagnation point flow. Coll. Surf..

[28] Dijt JC, Cohen Stuart MA, Fleer GJ (1994). Reflectometry as a tool for adsorption studies. Adv. Coll. Int. Sci..

[29] Nagle JF, Tristram-Nagle S (2000). Structure of lipid bilayers. BBA-Rev. Biomembranes.

[30] Lang H, Duschl C, Gratzel M, Vogel H (1992). Self-assembly of thiolipid molecular layers on gold surfaces: optical and electrochemical characterization. Thin Solid Films.

[31] Light Scattering From Polymer Solutions (ed. Huglin, M. B., Academic Press, 1972).

[32] Keller CA, Glasmästar K, Zhdanov VP, Kasemo B (2000). Formation of supported membranes from vesicles. Phys. Rev. Lett.

[33] Altschul SF (1997). Gapped BLAST and PSI-BLAST: a new generation of protein database search programs. Nucleic Acids Res..

[34] Sali A, Blundell TL (1993). Comparative protein modelling by satisfaction of spatial restraints. J. Mol. Biol..

[35] Laskowski RA, Macarthur MW, Moss DS, Thornton JM (1993). PROCHECK: a program to check the stereochemical quality of protein structures. J Appl Crystallogr..

[36] Wiederstein M, Sippl MJ (2007). ProSA-web: interactive web service for the recognition of errors in three-dimensional structures of proteins. Nucl. Ac. Res..

[37] Lindahl E, Hess B, van der Spoel D (2001). GROMACS 3.0: a package for molecular simulation and trajectory analysis. J. Mol. Model..

[38] Miyamoto S, Kollman PA (1992). Settle: An analytical version of the SHAKE and RATTLE algorithm for rigid water models. J. Comput. Chem..

[39] Shingate P, Sowdhamini R (2012). Analysis of domain-swapped oligomers reveals local sequence preferences and structural imprints at the linker regions and swapped interfaces. PLoS One.

[40] Hugonin L, Vukojević V, Bakalkin G, Gräslund A (2006). Membrane leakage induced by dynorphins. FEBS Lett..

[41] Balhara V, Schmidt R, Gorr S-U, DeWolf C (2013). Membrane selectivity and biophysical studies of the antimicrobial peptide GL13K. Biochim. Biophys. Acta.

[42] Li W (2015). Multimerization of a proline-rich antimicrobial peptide, Chex-Arg20, alters its mechanism of interaction with the *Escherichia coli* membrane. Chem. Biol..

