# Supplementary material for: An acidic model pro-peptide affects the secondary structure, membrane interactions and antimicrobial activity of a crotalicidin fragment
Source: Sci Rep. 2018 Jul 24;8:11127. doi: 10.1038/s41598-018-29444-0 (PMC6057973; doi:10.1038/s41598-018-29444-0)
Supplement: Supplementary file 1 — supplementary information [file 41598_2018_29444_MOESM1_ESM.docx]

An acidic model pro-peptide affects the secondary structure, membrane interactions and antimicrobial activity of a crotalicidin fragment

Supplementary Information

Nelson G. O. Júnior^1,2,3^, Marlon H. Cardoso^1,3,4^, Elizabete S. Cândido^1,4^, Daniëlle van den Broek^2^, Niek de Lange^2^, Nadya Velikova^5^, J. Mieke Kleijn^2^, Jerry M. Wells^4^, Taia M. B. Rezende^1,6^, Octávio Luiz Franco^1,3,4^, Renko de Vries^2*^.

*^1^Centro de Análises Proteômicas e Bioquímicas, Programa de Pós-Graduação em Ciências Genômicas e Biotecnologia, Universidade Católica de Brasília, Brasília-DF, Brazil*

*^2^Physical Chemistry and Soft Matter, Wageningen University, Stippeneng 4, 6708 WE Wageningen, the Netherlands*

*^3^Programa de Pós-Graduação em Patologia Molecular, Faculdade de Medicina, Universidade de Brasília, Brasília-DF, Brazil*

*^4^S-inova Biotech, Programa de Pós-Graduação em Biotecnologia, Universidade Católica Dom Bosco, Campo Grande-MS, Brazil*

*^5^Host-Microbe Interactomics, Wageningen University, P.O. Box 338, 6700AH Wageningen, the Netherlands*

*^6^ Curso de Odontologia, Universidade Católica de Brasília, Brasília-DF, Brazil.*

*^*^corresponding author, renko.devries@wur.nl*

**Maldi MS and MS/MS analysis of E_10_ Ctn[15-34] peptide**

MALDI MS analysis revealed that 30 min after dissolving the peptide E_10_ Ctn[15-34] in 10 mM Tris 100 mM NaCl, pH 7.5 and 10 mM K_2_HPO_4_ 50mM Na_2_SO_4_, pH 7.4 buffer, no modification of its mass (3662 Da) could be detected (Fig. SI-1A and B). For further confirmation, MS/MS analysis for the peptide was also performed, indicating the presence of 10 glutamic acids at the N-terminal portion, without the loss of a water molecule (18 Da loss). This means no pyroglumatization (cyclization) occurred for the first glutamic acid residue (Fig. SI-1C and D). The same analysis performed after keeping the peptide in the buffer at 37 °C for 24h, gave an identical result (Fig SI-2).

**Initial configurations for MD simulations**

*In silico* studies were carried out as described in the Materials and Methods section, to arrive at three dimensional structures for the peptides Ctn[15-34], E_10_-Ctn[15-34] and (GS)_4_-Ctn[15-34] to be used as initial configurations for the MD simulations. Molecular modeling simulations revealed that all peptides show three to four turns of α-helix at the N-terminus, and have a highly disordered coil conformations at the C-terminus (PMVIGVTIPF-NH_2_) (Fig. SI-3). From the modeling it also appears that the addition of pro-peptides (E_10_ and (GS)_4_) at the N-terminus seems (not taking in account any biomimetic environment) to enhance the helical content of Ctn[15-34] (Fig. SI-3B and C). Theoretical models for Ctn[15-34], E_10_-Ctn[15-34] and (GS)_4_-Ctn[15-34] were validated according to their fold quality, the models had z-scores in agreement to those observed for solution NMR structures deposited on PDB (Table SI-1). In addition, for all validated models, >90% of amino acid residues had angles within the most favorable regions in the Ramachandran plot, as well as overall g-factors above -0.5, suggesting they are indeed reliable structures to be used as initial configurations for our Molecular Dynamics simulations (Table SI-1).


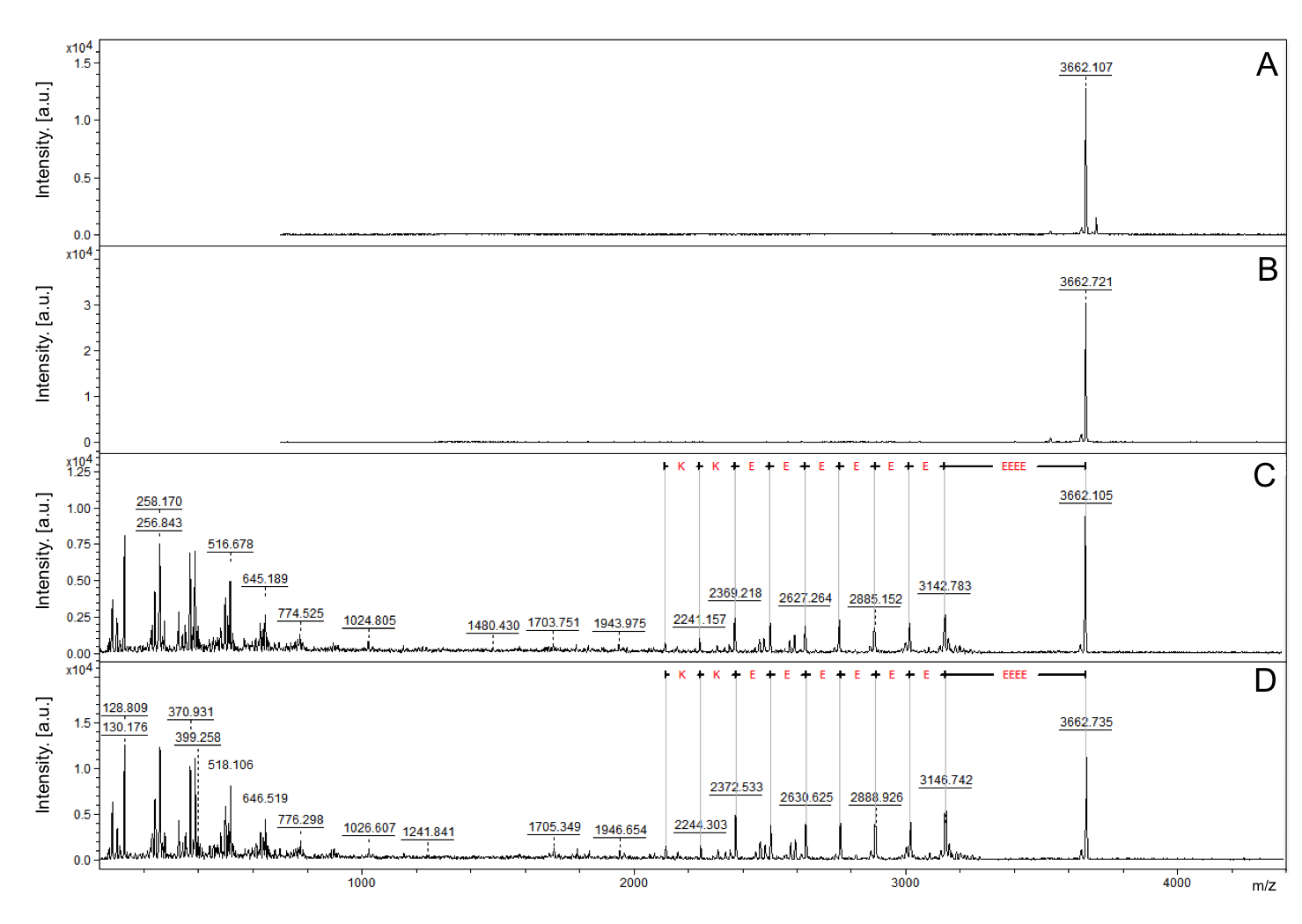


Fig. SI-1- MALDI-ToF MS and MALDI-ToF/ToF MS analysis for the peptide E_10_ Ctn[15-34]. A. MALDI-TOF MS spectra of peptide exposed to 30 min of 10mM potassium phosphate. B. MALDI-ToF MS spectra of peptide exposed to 30 min of 10mM Tris-HCl + 100mM NaCl. C. MALDI-ToF/ToF MS spectra of the ion m/z 3663.105 annotated the glutamic acid residues of N-terminal after 10mM potassium phosphate exposition. D. MALDI-ToF/ToF MS spectra of the ion at m/z 3663.735 annotated the glutamic acid residues of N-terminal after 10mM Tris-HCl + 100mM NaCl exposition.


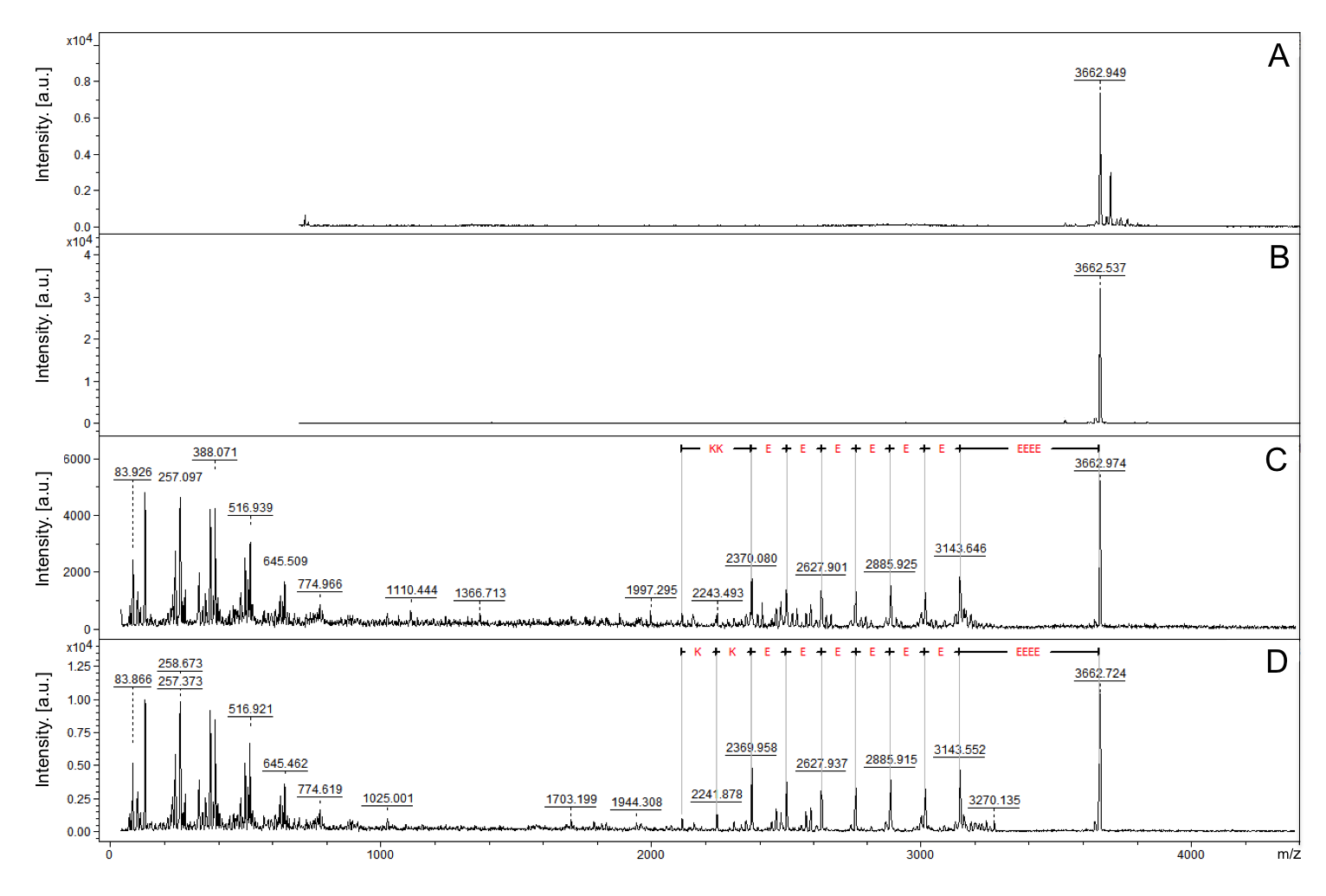


Fig. SI-2- MALDI-ToF MS and MALDI-ToF/ToF MS analysis for the peptide E_10_ Ctn[15-34] after 24h of exposition at 37°C. A. MALDI-ToF MS spectra of peptide exposed at 10mM potassium phosphate. B. MALDI-TOF MS spectra of peptide exposed at 10mM Tris-HCl + 100mM NaCl. C. MALDI-ToF/ToF MS spectra of the ion at m/z 3663.105 annotated the glutamic acid residues of N-terminal after 10mM potassium phosphate exposition. D. MALDI-ToF/ToF MS spectra of the ion at m/z 3663.735 annotated the glutamic acid residues of N-terminal after 10mM Tris-HCl + 100mM NaCl exposition.


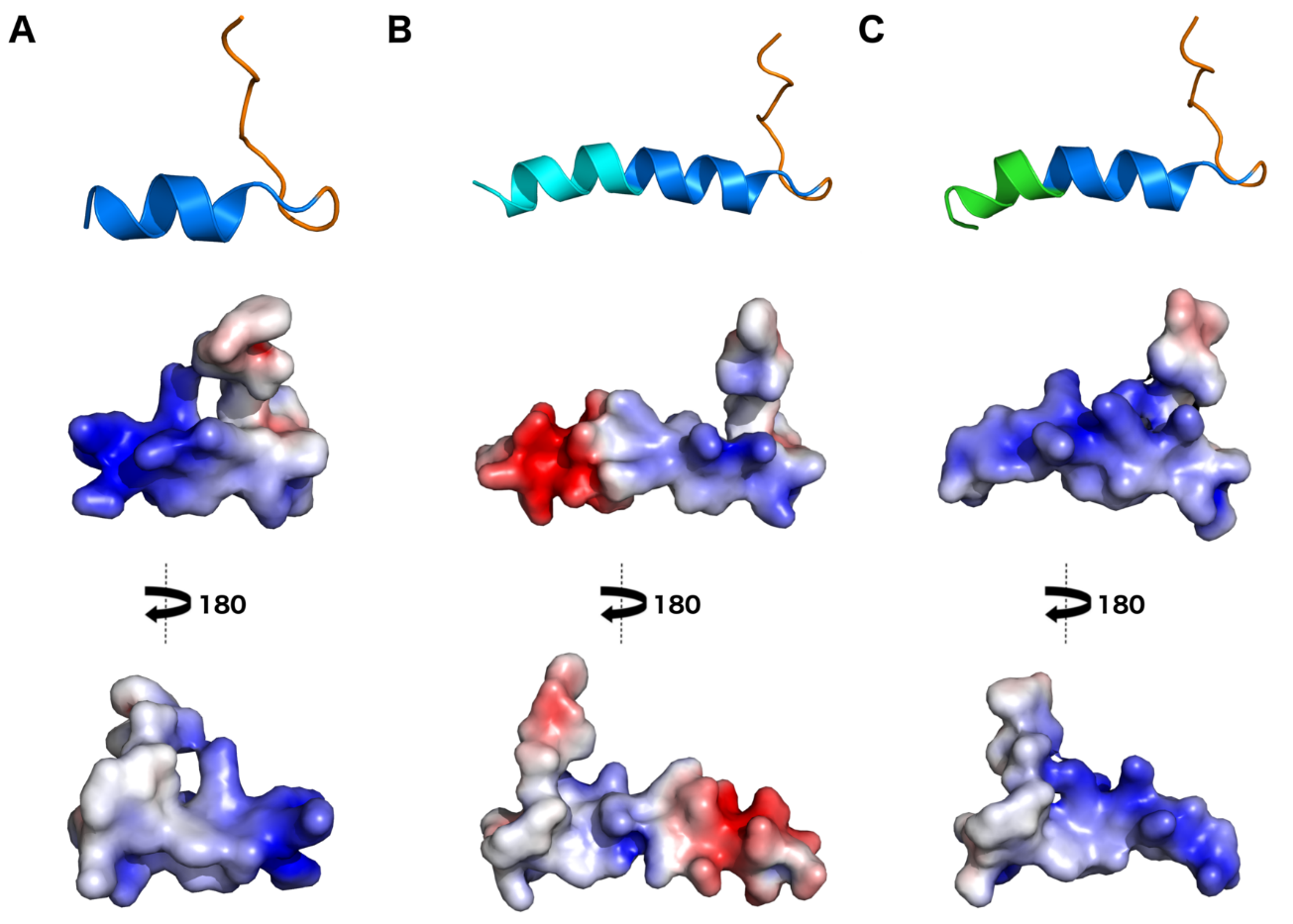


Fig SI-3 - Lowest free-energy three-dimensional theoretical models for (A) Ctn[15-34], (B) E_10_-Ctn[15-34] and (C) (GS)_4_-Ctn[15-34]. Adaptive Poisson-Boltzmann solver (APBS) electrostatic potential of (A) Ctn[15-34], (B) E_10_-Ctn[15-34] and (C) (GS)_4_-Ctn[15-34] ranging from − 5 kT/e (red) to + 5 kT/e (blue).

Table SI-1

| Peptide | ProSA-web (*z*-score) | PROCHECK (G-factor) | Ramachandran plot (most favorable region)% |
| --- | --- | --- | --- |
| Ctn[15-34] | -0.79 | 0.14 | 100 |
| E_10_-Ctn[15-34] | -1.07 | -0.07 | 96 |
| (GS)_4_-Ctn[15-34] | -0.9 | 0.09 | 90 |

Table SI- 1 - Folding quality and stereochemical validation of the three-dimensional theoretical models of Ctn[15-34] and its analogues
